# Are the Norms of Bayley Screening Test Appropriate for Persian Language Children?

**Published:** 2018

**Authors:** Farin SOLEIMANI, Nadia AZARI, Roshanak VAMEGHI, Firoozeh SAJEDI, Soheila SHAHSHAHANI, Hossein KARIMI, Adis KRASKIAN, Amin SHAHROKHI, Robab TEYMOURI, Masoud GHARIB, Nayereh MEHDIPOUR

**Affiliations:** 1 Pediatric Neurorehabilitation Research Center, University of Social Welfare and Rehabilitation Sciences, Tehran, Iran; 2 Department of Psychology, Karaj Branch, Islamic Azad University, Karaj, Iran; 3 Department of Occupational Therapy, Faculty of Paramedicine, Mazandaran University of Medical Sciences, Sari, Iran

**Keywords:** Developmental assessment, Infant, Child development, Testing norm, Screening tool, Child

## Abstract

**Objective:**

We aimed to assess the distribution of the Bayley screening test by age, and compare developmental risk category distributions between Persian language children and reference norms.

**Materials & Methods:**

A representative sample of 417 children, 1 to 42-months-old, by consecutive sampling from health -care centers were enrolled, during 2014 to 2015 in Tehran, Iran. The cognitive, language and motor development of children were evaluated using Bayley screening test. For determining cut-off point for the subtest scores, two cut-offs were determined for each age group, that classified children to the at risk, emerging, and competent categories. We estimated the agreement of the risk categories between the two samples using weighted kappa statistics.

**Results:**

About 70%-80% of all tests operated to the participating children were classified as normal by both norms. Weighted kappa coefficients for the five subtests ranged from 0.56 to 0.89 suggesting moderate agreement between two classification norms. Expressive and receptive communication had the lowest kappa scores (0.56 and 0.59, respectively), and classification of gross motor revealed the highest level of agreement (0.89).

**Conclusion:**

Developmental disabilities are common disorders that impose important functional limitations on the affected children. Identifying infants at risk for developmental disorders by screening is a main step to minimize complications. Dependence on reference-based norms for the Bayley screening test in Persian language children results in misclassification of risk category.

## Introduction

Developmental disorders are common problems that impose important functional limitations on developmental aspect of the affected children. More than 200 million children aged below 5 yr are not reaching their full potential for growth, and development due to biological and environmental risk factors (1). In the United States, 13% of 3-17 yr old children have a developmental disability and about 1.6% of children have global developmental delay (2). Health conditions such as low birth weight (LBW), preterm birth, perinatal infection, and birth defects increase the risk for developmental problems (3). In our background, asphyxia, LBW, preterm birth, and high-risk pregnancy have been shown to exert adverse effects on neurodevelopmental aspects (4-6).

In less developed countries, accessibility of adequate screening for developmental disorders is limited where costs on health are meaningfully lower than those in developed countries. 

Developmental disorders have a more damaging effect on health consequences in developing countries. Therefore determining at-risk infant for developmental abnormalities by screening tests is the main concern in less developed countries (7). 

The American Academy of Pediatrics recommends that pediatricians screen all infants and children for developmental problems during routine office visits (8). Developmental screening and early intervention maximizes a child’s ongoing functional abilities and helps to earn critical functional skills. However, without appropriate screening scale and relying on a clinician’s experience can be misleading in detection between normal and abnormal development (8, 9). 

Although it is necessary to evaluate appropriate growth and development in all children, there are only limited national data in the developing countries, because there are no locally developed psychometric tools, for detection of developmental delays and most extant findings were collected using tests created in developed countries. The usual method for test standardization is cross-sectional on healthy children of intended country. An important question is whether assessment tools that standardized in developed countries are applicable in less developed countries. 

In a study on 6150 infants, aged 1–18 months in Iran, the percentage of developmental delay varied depending on the considered cut-off point were reported, whereas, in referenced cut-off, 3.7% of study population had motor delay, and based on the Iranian cut-off points; it increased to 6.5% (10).

The Bayley screening test has been applied as a standard tool in developmental evaluation of infant in general pediatric populations, and it is particularly valuable in screening high-risk infants for developmental delay (11-13). This test briefly assesses cognitive, language, and motor developmental aspect of 1- 42 months old children. The period of test administration is short and a wide range of health practitioners with limited training can operate it. 

There are apprehensions about the use of a developmental assessment tool in other countries that the test was produced (14, 15). The following approaches are recommended for cross-cultural bias of using developmental assessment tools in a country, which developed in other countries. Firstly a new test formation can be done and normed for that population (16). Although, the new test is a culturally adapted scale, the construction of a test requires sufficient resources and can only be used in the population under study, thus limiting the comparison of the findings with other tests and between cultures.

An alternative way to the development of a new scale is the adaptation of existing tests for use in new country. Many guidelines have been published for this process (17,18). Although an adapted scale improves cultural suitability, it is also resource-intensive especially on language and cognition tests and does not permit comparability across countries.

The revision of existing assessment scales can reduce bias in test items of specific structures; there is a risk of deviation unless these adaptations are accompanied by local norms, because children in an environment can, on average, be better than other children owing to cultural differences in the education of children and access to elementary education (14, 15).

In this study, in order to evaluate the neurodevelopmental progress in Persian language children, we adapted and developed new norms to the Bayley screening test, and the distribution of developmental risk categories was compared with the norms of Tehran and the United States.

## Materials & Methods

A representative sample of 417, 1 to 42-months-old, healthy children by consecutive sampling from health -care centers recruited, in five geographical areas of Tehran city, Iran (north, south, west, east and center), during 2014 to 2015.

**Table 1:** Mean Scores of Bayley Screening Test by age

Age Group	Age (months)	Number	Mean Scores				
			cognitive	Receptive Communication	Expressive Communication	Fine Motor	Gross Motor
1	1-3	51	3.47	2.82	2.57	3.27	3.41
2	4-6	74	6.94	6.32	5.08	7.19	8.31
3	7-9	45	11.60	8.93	7.70	9.38	11.67
4	10-12	35	14.64	10.76	10.27	11.81	15.09
5	13-18	73	18.89	14.80	13.94	15.55	18.36
6	19-24	37	23.08	19.31	18.11	19.06	21.03
7	25-30	43	27.10	21.69	21.00	21.62	23.72
8	31-36	25	28.96	23.00	22.55	24.39	25.35
9	37-42	34	30.70	23.58	22.88	26.03	25.91

**Table 2:** Comparison of mean raw score using the United State and Tehran norms

Age (month)	Sample (Number)	Bayley Screening subtests (raw score)									
		Cognitive		Receptive Communication		Expressive Communication		Fine Motor		Gross Motor	
		Mean	t Value	Mean	t Value	Mean	t Value	Mean	t Value	Mean	t Value
2-4	Tehran (77)	5.16	0.193	4.70	1.320	4.15	2.019**	5.47	2.554**	6.19	2.630**
	US* (50)	5.10		5.10		4.70		4.70		5.30	
9-13	Tehran (69)	14.61	1.047	10.83	0.595	10.19	1.855	12.05	0.601	14.68	0.63
	US (51)	15.10		10.60		11.10		11.80		14.40	
19-26	Tehran (55)	24.10	1.930	19.98	2.888^**^	19.04	0.983	19.83	1.043	21.56	1.168
	US (52)	23.00		18.50		18.50		19.30		21.00	
33-42	Tehran (34)	30.44	1.767	23.50	1.256	22.69	1.647	25.66	0.373	26.72	0.815
	US (50)	31.30		23.20		23.40		25.50		26.40	

* United State

** *P*-value˂0.05

**Table 3:** Comparison of distributions of risk categories according to cut-off points of Tehran and United States

Bayley Screening subtests	Norms	Bayley screening test (risk categories)			Weighted Kappa (95% CI**)
		At Risk N (%)	Emerging N (%)	Competent N (%)	
Cognitive (n=396)	Tehran	10(2.5)	102(25.8)	284(71.7)	0.64*** (0.74-0.54)
	US*	5(1.3)	97(24.5)	294(74.2)	
Expressive communication (n=395)	Tehran	10(2.5)	89(22.5)	296(75.0)	0.59*** (0.69-0.49)
	US*	7(1.8)	68(17.2)	320(81.0)	
Receptive communication (n=392)	Tehran	7(1.8)	76(19.4)	309(78.8)	0.56*** (0.66-0.46)
	US*	6(1.5)	103(26.3)	283(72.2)	
Fine Motor (n=394)	Tehran	6(1.5)	97(24.6)	291(73.9)	0.67*** (0.77-0.57)
	US*	3(0.8)	85(21.6)	306(77.6)	
Gross motor (n=395)	Tehran	4(1.0)	84(21.3)	307(77.7)	0.89*** (0.99-0.79)
	US*	2(0.5)	74(18.7)	319(80.8)	

* United State

** Confidence Interval

*** P-value˂0.01

A healthy child was defined as any child born without congenital anomalies and medical complications and was not diagnosed with or receiving medication (including rehabilitation) for developmental disorders. The exclusion criteria were attention-deficit/hyperactivity disorder, chromosomal and genetic abnormalities, congenital infections, intellectual disability, inborn errors of metabolism, intraventricular hemorrhage, attachment disorder, sensory impairment, and Low Birth Weight.

The test was operated, by psychometrics, or child occupational therapists trained in the testing by the study investigators. The Bayley screening assesses skills of 1-42 months of age in cognitive, receptive and expressive communication, fine and gross motor domains. The main goal of this screening test is quick to determine whether a child progresses to normal expectations, and whether or not a more comprehensive assessment is needed. This test has been derived from the Bayley diagnostic test (11). This test consists of five sub-tests (each subtest contain 24 to 33 items). The total infant score after comparing with the norms is to classify the child's risk category of being competent, emerging, or at risk for developmental delay (11).

Two cuts points were determined for each age group, separating scores into the three bands that determine the at risk, emerging, and competent categories. Cut scores were based on age and represent approximately the 2^nd^ and 25^th^ percentiles. The 2^nd^ and 25^th^ percentile are the correspondings of 2 and 0.67 standard deviations below the mean, respectively.

The Bayley normative data were determined for 1700 American children with gestational age of 36–42 wk. Children with developmental disorder such as Down syndrome, cerebral palsy, pervasive developmental disorder, prematurity, language impairment or at risk of developmental delay constituted 10% of the sample (11). We used Persian version of Bayley screening test that psychometric properties (validity and reliability) was determined already (12, 13).

To determine the differences in scores by age, we compared the mean raw scores from the US and Tehran norms. Consistent to data available for the US norms (11), differences of scores in the Tehran and the US normative sample were compared for four age groups (2–4, 9–13, 19–26, and 33–42 months). We also categorized developmental class for each child according to both the US and Tehran norms. We estimated the agreement between the risk categories in two samples using weighted kappa statistics. 

Parents of all participants gave written informed consent. The Ethics Committee of the University of Social Welfare and Rehabilitation Sciences approved the research method. All parents were given age-specific developmental promotion guidance, independent of the child's ability. 

## Results

The sample included 417 children divided into 9 standard age groups. The sample was distributed evenly between male (*n* = 216, 51.8%) and female children (*n* = 201, 48.2%). Table 1 presents, number of children included in each age group, and mean raw scores of test by age. The results of the Tehran mean raw scores in comparison to the normative sample represent by Table 2. When review age groups, the significantly higher Tehran means scores in the fine and gross motor domains before 4 months; and at 19-26 months on receptive communication, domain determined.

The comparison of risk categories based on Tehran and US cut-off points represents by Table 3. Approximately 70%-80% of all tests results in classified children as normal by both norms. Weighted kappa coefficients for the five subtests ranged from 0.56 to 0.89 suggesting moderate to good agreement between the classification using the Tehran and US norms. Expressive and receptive communication subtests had the lowest kappa scores (0.56 and 0.59, respectively), and classification of gross motor demonstrated the highest level of agreement (0.89). 

## Discussion

Although evaluation of the risk factors on developmental aspect of children in developing countries is very important, majority of developmental assessment scales have originated from developed countries and have not been adopted in developing countries.

Our data show that the Bayley norms of children in Tehran are not equal to the reference norms, and using of the US standardized norms may result in misclassification of the functional developmental ability of our children. The relation between two norms was not the same, also was different on age groups and subscales.

These findings are predictable, because neurological development, especially cognitive domain related to factors such as; access to education, urbanization and economic situation (19-23).

Early childhood in the Asian background, compared to developed countries are characterized by different childcare actions for infants and children and different environmental capacity to health promotion and educational materials such as games, books, toys, and multimedia. Environmental and cultural background is likely to lead to differences in neurodevelopmental growth, especially in terms of language and cognition.

The risk classification of children in Tehran by the Bayley screening test was not discriminative by reference norms scores. We determine that the test efficiency differs between Tehran and reference sample, which our children having higher scores in the first 6 months of life and US children having higher scores after one-year-old. However, even when the normative means are similar, such as in the cognitive subtest, the differences in scores resulted in another neurodevelopmental risk category (weighted kappa = 0.64), signifying that different norms may be desired for all age groups.

Relating the neurodevelopmental risk category of the Tehran with the US cut-off points in our sample, we found meaningfully higher rates of at-risk infants in Tehran cut-off points compared to reference cut-offs. This may be related to the use of less strict inclusion criteria for healthy children in our sample, difference in cut-off points, or higher at-risk percentage in our sample compared to those in the US. On the other hand, the findings in recent studies in Western countries reveal the underestimation of developmental delay by the Bayley test (III edition) (24-32).

In the current study, the language scale (receptive and expressive communication) had the least correlation coefficient between two samples. Grammar and cultural adaptation and local norms are needed for using developmental assessments in countries with different language and child education practices. 

This is a first step in assessing the usefulness of the Bayley screening test in Persian language children. A major strength of this study was that we had to work the same statistical method used to create the US norms for this test. 

A limitation of our study was that our sample size (417 children in all age groups) may seem small compared to the 1700 US sample. Future studies must include a large sample of children demonstrative of the Persian language population to create local normative data, and additional studies are needed to assess efficiency of the Bayley scale properties among at-risk infants.

**In conclusion,** depending on the reference norms for the Bayley screening test in Persian language children results in misclassification of risk category of developmental delay. Thus, use of the Persian cut-off point is recommended for the exact identification of developmental risk category.
